# AMH and other markers of ovarian function in patients with Turner syndrome – a single center experience of transition from pediatric to gynecological follow up

**DOI:** 10.3389/fendo.2023.1173600

**Published:** 2023-06-29

**Authors:** Casper P. Hagen, Margit Bistrup Fischer, Gylli Mola, Theis Bech Mikkelsen, Line Hartvig Cleemann, Claus Højbjerg Gravholt, Mette H. Viuff, Anders Juul, Anette Tønnes Pedersen, Katharina Maria Main

**Affiliations:** ^1^ Department of Growth and Reproduction, Copenhagen University Hospital – Rigshospitalet, Copenhagen, Denmark; ^2^ International Center for Research and Research Training in Endocrine Disruption of Male Reproduction and Child Health (EDMaRC), University of Copenhagen, Rigshospitalet, Denmark; ^3^ Department of Molecular Medicine, Aarhus University Hospital, Aarhus, Denmark; ^4^ Department of Diabetes and Endocrine Diseases, Aarhus University Hospital, Aarhus, Denmark; ^5^ Department of Clinical Medicine, University of Copenhagen, Copenhagen, Denmark; ^6^ Department of Gynecology, The Fertility Clinic, Copenhagen University Hospital – Rigshospitalet, Copenhagen, Denmark

**Keywords:** ovarian function, fertility preservation, turner syndrome, anti mullerian hormone (AMH), FSH (Follicle Stimulating Hormone), inhibin B

## Abstract

Turner syndrome (TS) is a chromosomal disorder that affects about 1 in 2500 female births and is characterized by the partial or complete absence of the second X chromosome. Depending on karyotype, TS is associated with primary ovarian insufficiency (POI). Approximately 50% of girls with a mosaic 45, X/46, XX karyotype may enter puberty spontaneously, but only 5-10% of women with TS achieve pregnancy without egg donation. In this review, we will evaluate the clinical use of markers of ovarian function in TS patients. Based on longitudinal studies of serum concentrations of reproductive hormones as well as ovarian morphology in healthy females and patients with TS, we will evaluate how they can be applied in a clinical setting. This is important when counseling patients and their families about future ovarian function essential for pubertal development and fertility. Furthermore, we will report on 20 years of experience of transition from pediatric to gynecological and adult endocrinological care in our center at Rigshospitalet, Copenhagen, Denmark.

## Introduction

Various pathological conditions cause early loss of ovarian follicles resulting in absence or cessation of pubertal development and primary or secondary amenorrhea (premature ovarian insufficiency, POI). The most prevalent inherited condition of accelerated follicle loss is Turner syndrome (TS) affecting approximately 1:2500 liveborn females (1).

**Figure 1:**
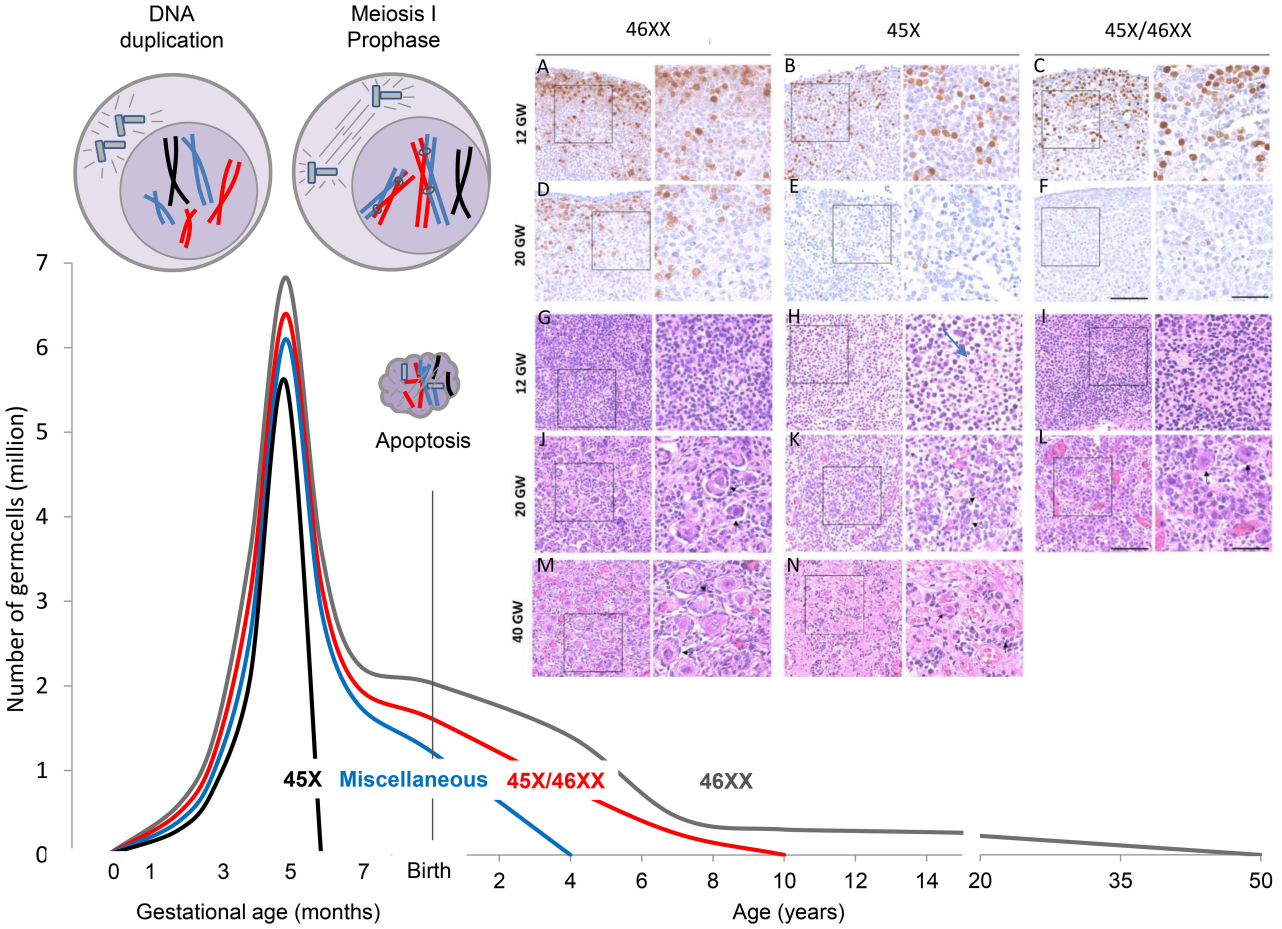
Accelerated loss of follicles depends on the TS karyotype. The mechanism is believed to be apoptosis caused by pairing failure of homologous chromosomes in meiosis I. This is schematically shown in the top left corner with only one duplicated X chromosome (black). Histology samples **A-N** modified from (5): In early fetal life, there are plenty of OCT4 positive oogonia present in 45, X ovaries **(B)**, but many of the germ cells are degenerated with contracted nuclei and a thin layer of cytoplasm (**H** arrow). Later in gestation, primordial and small growing follicles are present in the healthy ovary (J+M), whereas somatic cells and fibroblasts are abundant in the 45, X ovary (K+N). Schematic illustration of the number of germ cells in healthy females (46,XX, grey line) from early fetal life to time of menopause; data based on Baker et al. (6, 7).

Due to complete or partial loss of one X-chromosome in all cells (e.g. 45,X) or part of the cells (mosaicisms, e.g. 45,X/46,XX), TS patients suffer from a variable degree of prenatal loss of follicles (2–5) (**Figure 1**).

When TS is diagnosed during childhood, patients and their families are often concerned about future reproductive potential. Will they develop similar to their teenage peers? Will they enter puberty spontaneously without hormone replacement therapy? Will they eventually achieve pregnancy? The increasing success rates of ovarian cryopreservation for future fertility in girls with cancer prior to gonadotoxic therapy have inspired similar protocols in patients with TS. In experimental settings, cryopreservation of ovarian tissue has been performed, and it is essential only to offer cryopreservation to patients with ovarian follicles (8, 9).

**Figure 2:**
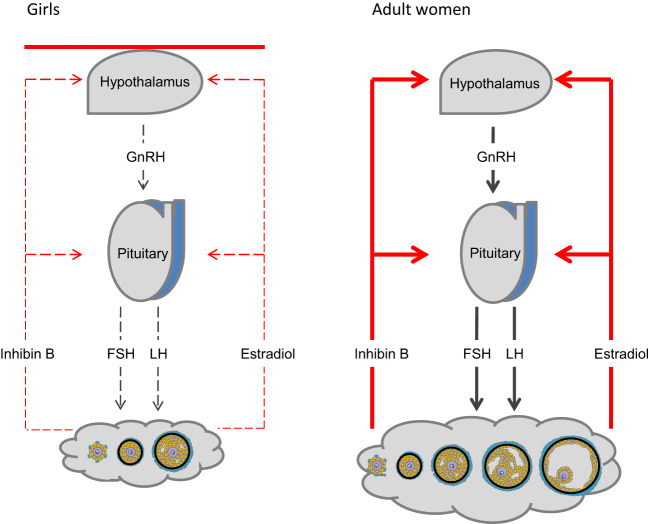
The hypothalamic-pituitary-gonadal (HPG) axis. Activity of the hypothalamus and pituitary is regulated by negative feedback of ovarian hormones (right). The HPG axis is centrally inhibited during mid-childhood (left). Follicles are primarily restricted to stages growing independently from FSH stimulation. Only occasionally FSH- induced follicle growth occurs.

However, it is a challenge to assess ovarian activity in girls and it is even more difficult to predict future ovarian function. Apart from a transient neonatal gonadotropin surge, the hypothalamic-pituitary-gonadal (HPG) axis is quiescent until pubertal onset allowing only gonadotropin-independent growth of follicles reaching small antral stages. Therefore, in TS patients with streak ovaries, the usual lack of negative feedback and consequently hypergonadotropic hypogonadism is not evident prior to time of expected pubertal onset (8–15)(**Figure 2**).

Today, the best candidate as a marker of subtle ovarian activity is Anti-Müllerian Hormone (AMH) produced by granulosa cells in small growing follicles (11). Initially, the focus of attention on this peptide was the testicular production of AMH. Alfred Jost was the first to suggest that a substance produced from the developing gonad in the male fetus was responsible for the regression of the Müllerian ducts (ovarian ducts, uterus and the proximal one-third of the vagina) (12). This hormone is AMH, previously referred to as Müllerian Inhibiting Substance (MIS), produced by immature Sertoli cells in the male fetus (13, 14). AMH is a member of the TGF-beta family. It is encoded by the *AMH* gene (15) which is located on chromosome 19p13.3 (16). AMH exerts its effect through the single transmembrane receptor, AMH type 2 (AMHR2), leading to phosphorylation of Smad 1/5/8 that enter the nucleus and regulate transcriptional activity (17). In young patients with Differences of Sex Development (DSD), high serum concentration of AMH is a specific and sensitive marker of testicular tissue (immature Sertoli cells) in the gonad (18–21).

**Figure 3:**
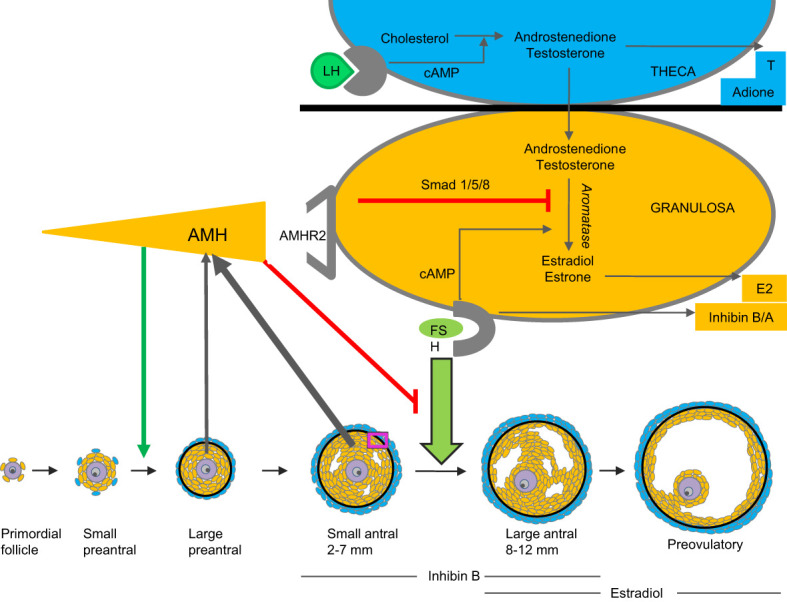
Model of AMH production and action. AMH is produced by granulosa cells of small growing follicles. It inhibits FSH-induced follicle growth as well as gonadotropin-induced aromatization of androgens to estrogens (9, 24–26).

In females, circulating AMH originates exclusively from the ovaries (22). The function of AMH is not fully elucidated but knock-out mice models and human *in vitro* data indicate that AMH inhibits follicle growth as well as FSH induced aromatase activity (11, 23–25). Effects on recruitment from primordial follicles may be species dependent. AMH promotes primordial follicle recruitment in cultured human ovaries (9) and *in vitro* and *in vivo* data from non-human primates support stimulating action of AMH on preantral follicle growth (26) (**Figure 3**). Thus, production and effects of AMH are follicle stage dependent and AMH seems to play an essential role as gate-keeper for FSH-induced follicle maturation, estradiol production, as well as regulator of the selection of the dominant follicle in the late follicular phase of the menstrual cycle. In humans, rare mutations of the gene encoding *AMH* result in premature ovarian insufficiency (27). Extragonadal effects of AMH have been proposed, and AMH may play a role in upregulation of GnRH dependent LH pulsatility (28). Circulating AMH levels are usually elevated in PCOS patients as well as in patients with granulosa cell tumors (29–31).

**Figure 4:**
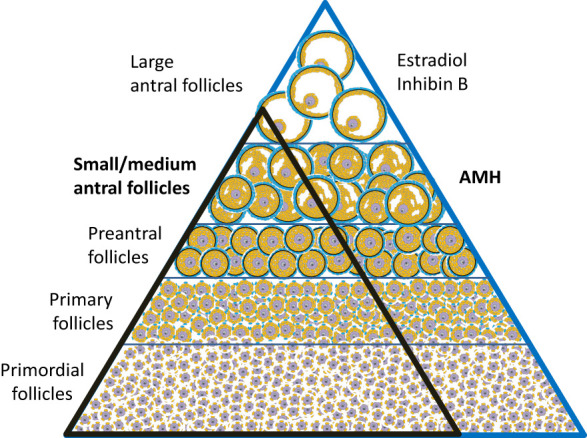
The number of follicles decline as they mature. Histological studies suggest that the number of follicles in different stages is in equilibrium; i.e. an individual with many primordial follicles have more preantral and antral follicles (blue triangle) compared with an individual with fewer primordial follicles (black triangle) (38, 39).

The unique source of AMH from follicles growing independently of FSH-stimulation poses several clinical advantages. Circulating AMH levels are more refractive to fluctuations of gonadotropin levels compared to hormones produced by larger follicles. Thus, circulating concentrations of AMH are relatively stable through the menstrual cycle (although cycle dependent fluctuations are more pronounced in women with higher AMH concentrations) (32–34). AMH is decreased app. 30% by oral contraceptive therapy (35) and 50% during pregnancy (36). In healthy adult women, serum levels correlate with the number of antral follicles (37). Due to a fine equilibrium between follicles in different stages (38, 39), AMH levels reflect the number of primordial follicles constituting the ovarian reserve (40) (**Figure 4**). In healthy adult women, circulating AMH is therefore predictive of the reproductive lifespan (41–45). Women with age specific low AMH tend to enter menopause earlier than women with higher AMH. However, considerable overlap exists, and the predictive value for AMH in a given woman concerning age at menopause is limited (46).

In this review, we will present data relevant when assessing AMH in girls and adolescents with TS. To interpret a given AMH measurement in a patient at risk of POI, it is essential to know details about AMH in healthy girls. Age specific reference ranges are mandatory. Additionally, cross sectional studies of AMH in relation to ovarian morphology are necessary to assess if AMH in girls reflects the number of small antral follicles – which may reflect the ovarian reserve of primordial follicles. Longitudinal studies of individual AMH levels are needed to evaluate the predictive value of AMH concerning future ovarian activity in healthy girls as well as in patients with TS. Further, we will briefly discuss the qualitative aspect of AMH concerning fecundability.

## Karyotype as predictor of ovarian activity

In TS patients, the karyotype is strongly associated with ovarian status; i.e. the risk of POI is highest in monosomic patients compared to karyotypes with mosaicism including a healthy cell line (45,X/46,XX) or isochromosomes (6, 47–51). The mechanism causing accelerated loss of germ cells is believed to be apoptosis caused by pairing failure of homologous chromosomes in meiosis I. In early fetal life when the first oocytes enter the diplotene stage of meiotic prophase I, there are plenty of oogonia present in 45,X ovaries (**Figure 1**, histology section B, OCT4), but many of the germ cells are degenerated with contracted nuclei and a thin layer of cytoplasm (**Figure 1**, arrow in section H). Later in gestation, primordial and small growing follicles are present in the healthy ovary (**Figure 1**, J+M), whereas somatic cells and fibroblasts are abundant in the 45,X ovary (**Figure 1**, K+N). There are very few follicles.

In theory, the loss of follicles depends on the specific TS karyotype: Patients with 45,X are often born with streak gonads (**Figure 1**, black line) whereas TS patients with mosaicisms including a healthy cell line (45,X/46,XX) have approximately 50% chance of entering puberty spontaneously (**Figure 1**, red line) (6). All other TS genotypes caused by structural abnormalities of one X chromosome are referred to as miscellaneous having intermediate chance of preserved ovarian function (**Figure 1**, blue line).

The degree of mosaicism evaluated in 30 white blood cells may not be fully representative of the gonadal mosaicism (52). That is also the case when patients are diagnosed prenatally by non-invasive prenatal testing, amniocentesis, chorionic villus sampling or by fetal DNA in maternal blood sample. Furthermore, different tissue from the same patient – and even different cells from the same ovary may express variable degree of mosaicism (53, 54). Thus, the proportion of affected cells in peripheral blood is not always predictive of the remaining primordial follicles. This may explain cases of apparently monosomic patients with preserved ovarian function (55). There are even reports of 45,X patients with multiple unassisted pregnancies (56). Patients with miscellaneous karyotypes have an intermediate chance of maintaining ovarian activity, but from the limited number of patients with specific genotypes, it is not possible to clarify if certain loci are more prone for POI than others. Patients with TS including Y chromosome material are at risk of developing gonadoblastoma, and gonadectomy is recommended, although the degree of risk of gonadoblastoma still remains to be firmly established.

Thus, the karyotype based on DNA from white blood cells can be misleading concerning the degree of ovarian dysgenesis. The karyotype is a strong indicator of the degree of ovarian dysgenesis, but additional markers are needed to evaluate the ovarian function of girls and adolescents with TS.

## Reproductive hormones

**Figure 5:**
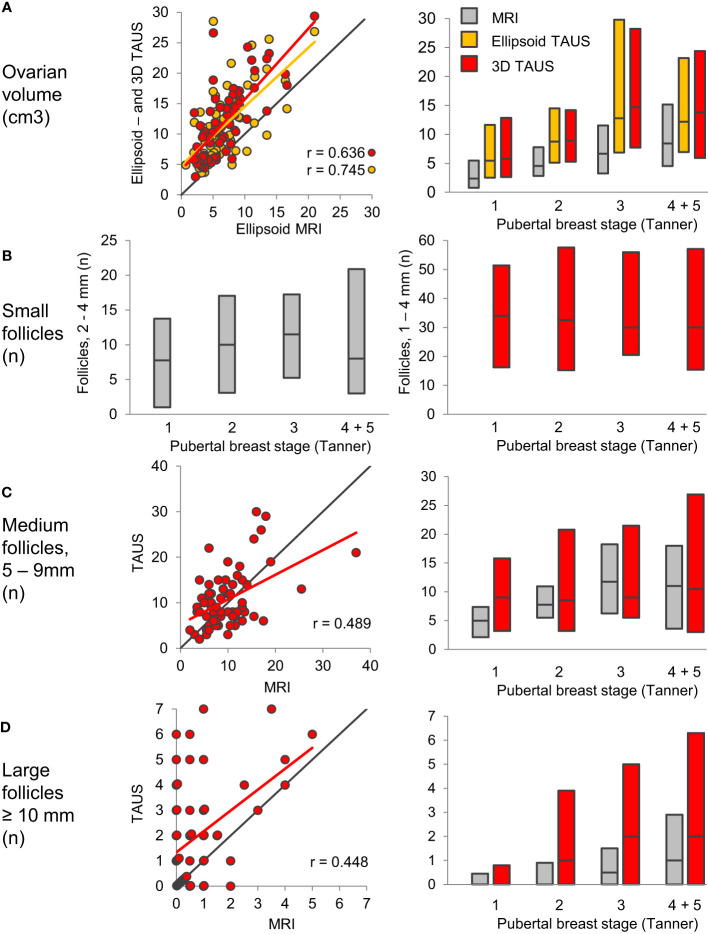
Ovarian volume **(A)** and follicle numbers **(B–D)** assessed by MRI (grey) and TAUS; Ellipsoid TAUS (orange) and 3D TAUS (red), according to pubertal breast stages. Bars indicate median, 10th and 90th percentiles. Black lines indicate lines of identity. Figure from (57).

**Figure 6:**
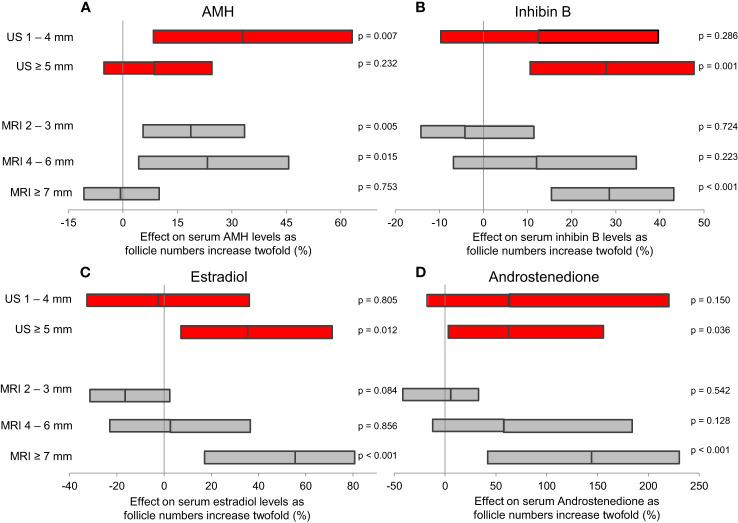
Results of multiple regression analyses evaluating which follicles contributed most to serum levels of ovarian hormones. The estimated effect of a two-fold increase (doubling) in follicle numbers is presented as geometric mean (95% CI). Both TAUS (red) and MRI (grey) revealed that AMH **(A)** is produced by smaller follicles than inhibin B **(B)**, estradiol **(C)** and androstenedione **(D)**. Figure from (57).

Detailed magnetic resonance imaging (MRI) and trans-abdominal ultrasound studies (TAUS) of ovarian follicle numbers in healthy girls revealed that small antral follicles were present in all prepubertal girls (57). Large follicles were present after pubertal onset, and the number of large follicles increased as puberty progressed (**Figure 5**). This knowledge of ovarian morphology is important for interpreting circulating levels of reproductive hormones during childhood. Pubertal reactivation of the HPG axis and increasing levels of gonadotropins is essential for maturation of follicles into large antral stages responsible for steroid hormone production. Thus, inhibin B and estradiol (produced by granulosa cells) as well as testosterone and androstenedione (produced by theca cells) correlated strongly with the number of large follicles (57), independent of pubertal stages (**Figures 6B–D**).

**Figure 7:**
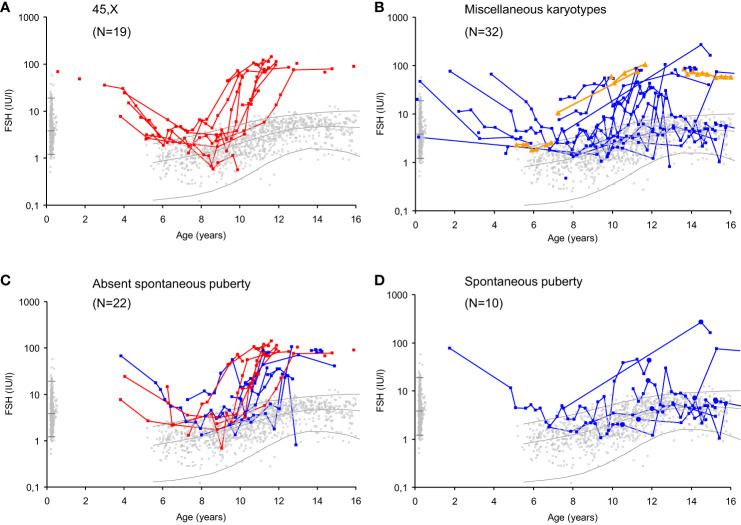
Serum FSH levels (IU/l) in girls with Turner syndrome (n=51) according to age, karyotype and spontaneous puberty onset compared to a reference range based on 2406 healthy Danish girls (grey dots). Lines represent geometric means and 95% prediction interval ( ± 2SD). Girls with 45,X monosomy (red, **(A)**; miscellaneous TS karyotypes before (blue) and after gonadectomy (orange) **(B)**; TS patients with absent spontaneous puberty **(C)** and spontaneous puberty **(D)**. Age at spontaneous pubertal onset is illustrated by closed circles. Figure from (47).

These morphological findings and their association with hormone levels explain the clinical challenges the pediatrician faces when evaluating ovarian activity during the quiescence of the HPG axis in mid-childhood. Reproductive hormone levels in mid-childhood are therefore similar to healthy girls; i.e. low levels of LH and FSH from the pituitary as well as low or undetectable levels of inhibin B and estradiol produced by granulosa cells surrounding larger antral follicles (8, 9, 58). Centrally inhibited levels of FSH, albeit measurable, are rarely sufficient for follicle maturation beyond small antral stages (**Figure 2**). Thus, in our longitudinal study of reproductive hormone levels in TS patients through childhood, gonadotropins were not elevated in the majority of patients who did not enter puberty spontaneously (FSH data seen in **Figure 7**) (47). However, there are indications that HPG activity during minipuberty does not end as abruptly in girls as in boys. Thus, FSH seems to be elevated in young prepubertal Turner syndrome patients up to 6 years of age (47, 58, 59). A single measurement of undetectable inhibin B was a prevalent finding in healthy girls and therefore not a very specific predictor of absent pubertal onset in TS patients. However, repeated blood samples increased the chance of revealing ovarian activity by detecting inhibin B produced by a randomly matured large follicle (47).

Introduction of ultra-sensitive liquid chromatography–mass spectrometry (LCMS/MS) indicates that estrone (E1) is measurable in the majority of healthy prepubertal girls (10). Further studies on circulating concentrations of estrone and estradiol (LCMS/MS) in girls with TS are needed to evaluate the predictive value of these biomarkers. Despite ultra-sensitive LCMS/MS methods enabling measurement of low levels of circulating androgens, these hormones are co-produced by the adrenals and therefore not specific for ovarian activity (60).

Even after spontaneous pubertal onset and/or menarche, it remains a clinical challenge to evaluate ovarian function. Irregular anovulatory cycles are prevalent in healthy girls up to 2-3 years after menarche (61). Furthermore, reproductive hormones may remain within the normal range before POI is clinically evident, despite significant depletion of the ovarian reserve (37, 62, 63).

Thus, during mid-childhood, the clinical use of gonadotropins and products from larger ovarian follicles (inhibin B, estradiol, testosterone and androstenedione) is hampered by central inhibition of the HPG axis.

However, in clinical follow up, repeated assessments prior to pubertal onset may reveal ovarian activity (detectable inhibin B levels) or hypergonadotropic hypogonadism (elevated FSH levels).

## AMH in healthy girls

**Figure 8:**
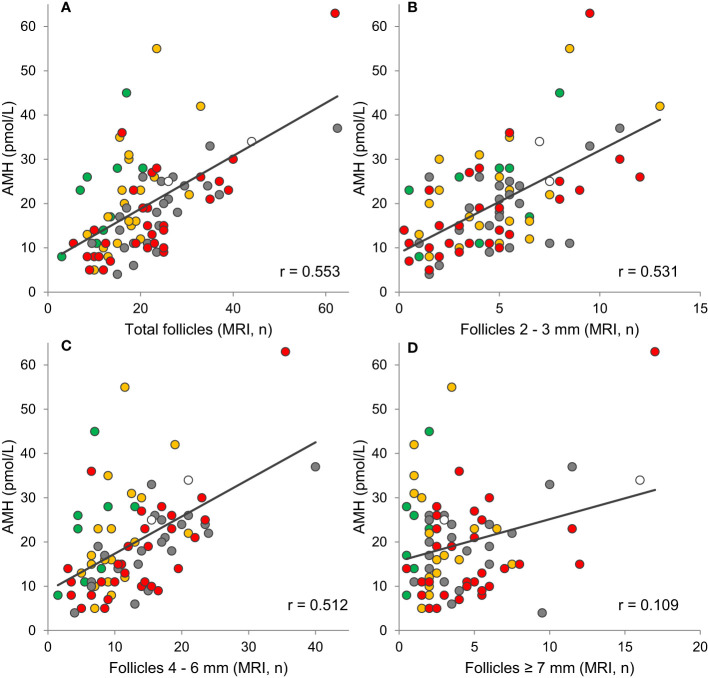
Correlations between AMH and **(A)** the total number of follicles (r=0.553, p<0.001), **(B)** small follicles (r=0.531, p<0.001), **(C)** medium follicles (r=0.512, p<0.001), and **(D)** large follicles (r=0.109, p=0.323) evaluated on MRI. Tanner stage B1, orange: B2, grey: B3, red: B4 + 5, white: Tanner stage unknown. Grey lines indicate tendency lines for correlations including all samples. Figure modified from (57).

Interestingly, circulating AMH reflects the number of small and medium antral follicles in healthy peripubertal girls (57) (**Figures 6A**, **8**). Thus, AMH is a unique marker of ovarian activity during mid-childhood quiescence of the HPG axis.

**Figure 9:**
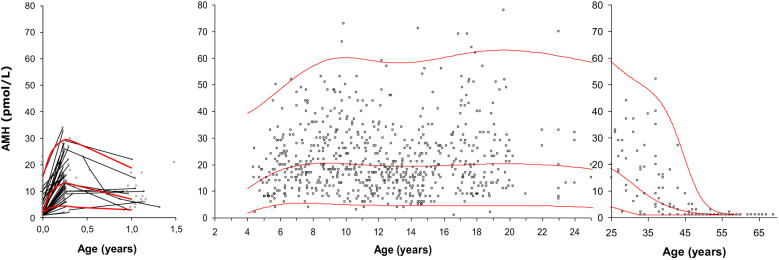
Serum AMH (pmol/L) in 926 healthy infants, girls, adolescents and adult women. Longitudinal values during infancy are connected with black lines. The red curves represent the median, the 2.5^th^ percentile and the 97.5^th^ percentile. Figure modified from (64).

We established the first reference range of AMH in females measured with a sensitive assay. It was based on 926 healthy females from birth to 69 years of age (**Figure 9**). We observed a surge of AMH at time of the so-called “minipuberty” (the transient postnatal activation of the HPG axis) (64). This was confirmed in a recent detailed longitudinal study of healthy girls – even indicating a biphasic pattern of AMH and other reproductive hormones during the first year of life (65).

The transient stimulation of the ovaries during minipuberty results in increasing numbers of antral follicles producing AMH (66). AMH seems to increase from 4 to 8 years of age, but compared to other reproductive hormones, circulating levels of AMH are remarkably stable in childhood, puberty and adolescence (64, 67). However, inter-individually between girls, AMH levels vary 15-fold. These findings are in line with the dynamics of ovarian follicles, as the number of AMH-producing follicles (antral follicles < 6mm) varies between healthy peripubertal girls but the number of these small growing follicles do not increase after pubertal onset (57).

A recent long-term longitudinal study of healthy females followed from infancy to adolescence reveal remarkable stable levels of AMH through the entire childhood (68). If a girl had high AMH, she remained with high levels through infancy, childhood, puberty and adolescence, and vice versa if she had low levels, she maintained low levels. Thus, the predictive value of low concentration of circulating AMH in mid-childhood is both sensitive and specific of low AMH in adolescence. Due to individual tracking of activity from small growing follicles, AMH in mid-childhood - and even in infancy - was associated with the number of small follicles in the same girl at puberty and adolescence. A meta-analysis including data from several studies suggests that AMH increases in late adolescence (69). The study is based on data from different cohorts using different immunoassay which are difficult to convert to comparable levels (70, 71). Circulating AMH is present in different molecular forms (72) which may explain the discrepancy between AMH assays (73). There is a need of an international standard to enable comparison of AMH levels between study populations when measured at different laboratories.

Thus, in healthy girls, AMH is a unique reproductive hormone reflecting and predicting the number of small antral follicles. Individual circulating levels are stable through infancy, childhood, puberty, and adolescence.

## Regulation of AMH

**Figure 10:**
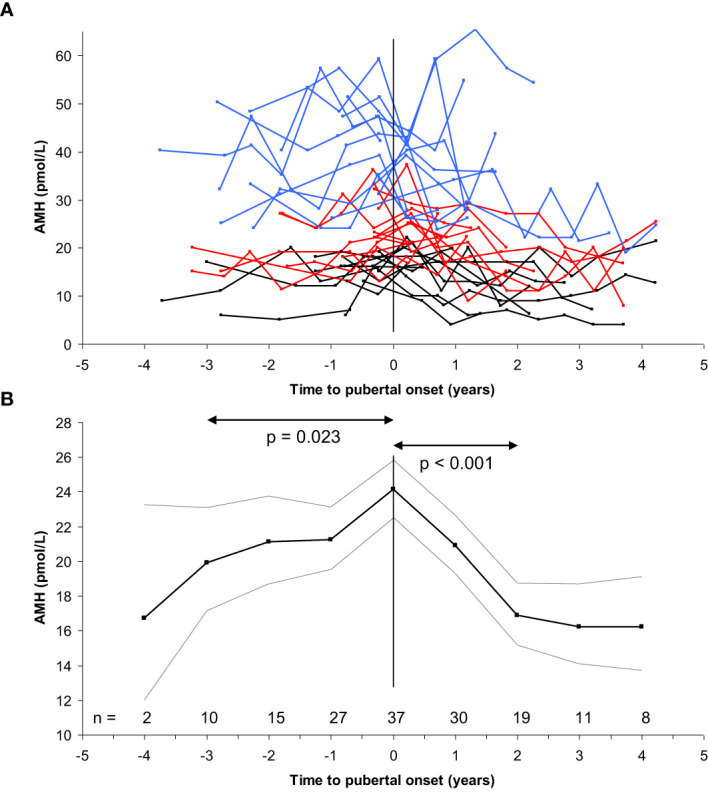
**(A)** Longitudinal AMH levels in 39 girls according to time to pubertal onset (breast development) (top). Blue, red and black lines indicate highest, medium and lowest tertile of AMH, respectively. **(B)** Variance component model (bottom) of the longitudinal data (black line: geometric mean, grey lines: +/- 1.96SD) revealed minor increase of AMH levels during the three years prior to pubertal onset (20 to 24 pmol/L, 17%, p=0.023), followed by decreasing levels two years after pubertal onset (24 to 17 pmol/L, 30%, p<0.001). Subsequently, AMH levels were stable. Figure based on data from (74).

In healthy girls, circulating AMH levels are negatively associated with FSH levels prior to pubertal onset (74). Furthermore, detailed longitudinal data revealed a limited but significant increase of AMH prior to pubertal onset (+17%) followed by decreasing levels (-30%) two years after pubertal onset. These findings have been confirmed by two British cohorts of healthy peripubertal girls (75, 76) (**Figure 10**). Initially, we speculated that the post-pubertal decrease of AMH was caused by the pubertal increase of FSH, leading to increased maturation of follicles which would reduce the number of AMH producing follicles. However, our detailed study of ovarian morphology revealed that the number of AMH producing follicles (< 6mm) actually increased during early puberty (57). In the same study, independent of follicle numbers, estradiol levels were negatively correlated with AMH. Increasing estradiol during early puberty may therefore directly inhibit AMH production. Firm causal conclusions of the negative association between AMH and FSH as well as estradiol cannot be drawn from our human clinical data. However, direct inhibition of AMH expression by estradiol has been suggested by *in vitro* studies of granulosa cells from patients undergoing *in vitro* fertilization (77). Conversely, AMH reduces sensitivity and growth rate of follicles in response to FSH as well as inhibits aromatase expression in smaller follicles (11, 26, 78). Thus, AMH seems to inhibit estradiol production in small follicles, whereas estradiol may inhibit AMH production in large follicles. We have speculated that in prepubertal girls, AMH is essential to prevent FSH-induced growth as well as premature estradiol production from small growing follicles.

The data discussed above are from healthy girls with an intact HPG axis. Cellular studies suggest that FSH does not affect AMH production in granulosa cells from healthy women (79), however, these studies were performed on granulosa cells retrieved from ovarian stimulation which may affect the response. Further insight in regulation of AMH is gained from studies manipulating the HPG axis. From small cross-sectional studies of women on hormonal contraceptive treatment (HCT), AMH levels were considered independent of pituitary activity (36, 80). However, larger cross-sectional studies as well as recent longitudinal studies suggest that AMH levels are reduced app. 30% by HCT (35, 81, 82). Whether this is caused by direct inhibition of AMH expression by estradiol or the effect is due to reduced number of medium antral follicles caused by suppression of GnRH secretion by potent synthetic estrogens remains to be elucidated. In our study of AMH levels in girls with central precocious puberty before, during and after GnRH agonist treatment, AMH was reduced 50% in response to suppression of pituitary activity (83). Although ultrasound was not performed on these girls, previous studies suggest reduced number of medium sized antral follicles during GnRHa treatment (84). This would be a plausible explanation for our findings.

In conclusion, the negative correlation between AMH and FSH supports that a degree of negative feedback between pituitary gonadotropin secretion and ovaries is exercised even in prepubertal girls.

## AMH as a predictor of fecundability in adult women

**Figure 11:**
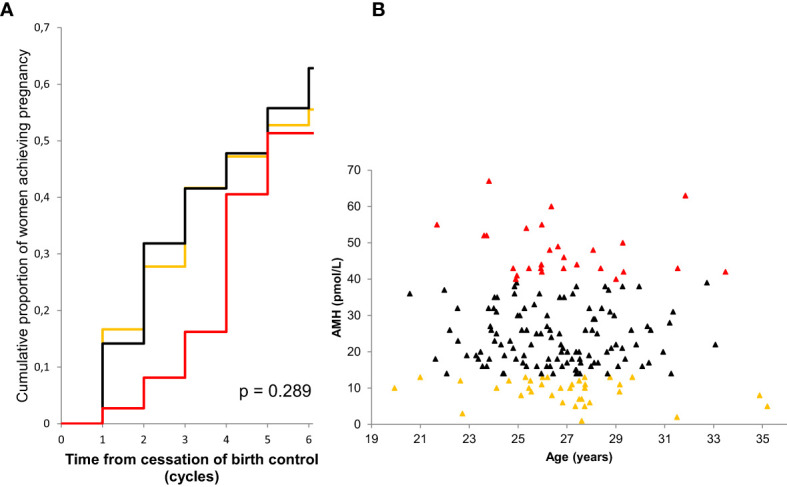
Kaplan-Meier curves showing the cumulative proportion of pregnancy by serum level of AMH **(A)**. Low AMH (quintile 1) orange line, medium AMH (quintiles 2-4) black line, high AMH (quintile 5) red line. P-value describes difference between curves (log-rank test). AMH as a function of age in 186 participating women **(B)**. Colors correspond to subgroups of AMH levels: low (orange), medium (black) and high (red). Figure from (92).

Whereas the value of AMH as a quantitative marker of follicles seems to be established, it remains contentious whether AMH is a marker of oocyte quality. Data from IVF settings strongly suggest circulating AMH as a marker of oocyte quality. AMH predicts the ovarian response (85, 86), and positive associations with the chance of conception (87) and livebirth (88, 89) have been reported. However, data from healthy women are less convincing. The first report of AMH as a marker of fertility in healthy women indicated that very low AMH predicted reduced fecundability in 100 women in their late reproductive life (30 – 42 years) (90). In another study of sub-fertile women who were unsuccessful in conceiving after 12 months of unprotected sexual intercourse (mean age 36 years), AMH levels in the 14 women achieving pregnancy during the following 6 months were not different from the 69 non-pregnant women (91). In a large prospective study of 186 healthy women (mean age 27 years) adjusted for male confounders, we found that high but not low AMH predicted reduced fecundability (**Figure 11**) (92). Our finding that high AMH was associated with reduced fecundability is most likely explained by a PCOS-like biochemical profile in the females with high AMH. The low AMH tertile included women witih AMH < 13 pmol/L which is well above the detection-limit of the assay (2 pmol/L) and the -2SD of the reference range in young adults (5 pmol/L). Thus, the size of the study population did not allow us to evaluate the effect of very low AMH. In support of our findings, a study of 1202 healthy women who had previously conceived did not find a reduced fecundability in women with low AMH (93). There is the possibility that sub-fertile PCOS patients may have been excluded in the study which may explain why high AMH was not associated with reduced fecundability in their cohort. In another study, AMH levels measured in the first trimester of pregnancy was not associated with fecundability (self-reported) in a retrospective study of 87 healthy women conceiving naturally (mean age 31 years) (94). Other cohorts of different ethnicity support that low AMH is not associated with reduced fecundability (95).

Patients with TS have increased risk of autoimmune conditions, and untreated Hashimoto´s hypothyroidism may contribute to reduced fecundability in adult patients with preserved ovarian function.

In conclusion, AMH in adult women seems to be a quantitative rather than a qualitative marker of ovarian follicles. Further studies are necessary to elucidate if extremely low AMH affects time to pregnancy and to confirm whether low AMH predicts reduced fecundability in healthy women in late reproductive life.

## AMH as marker of ovarian activity in ts patients

**Figure 12:**
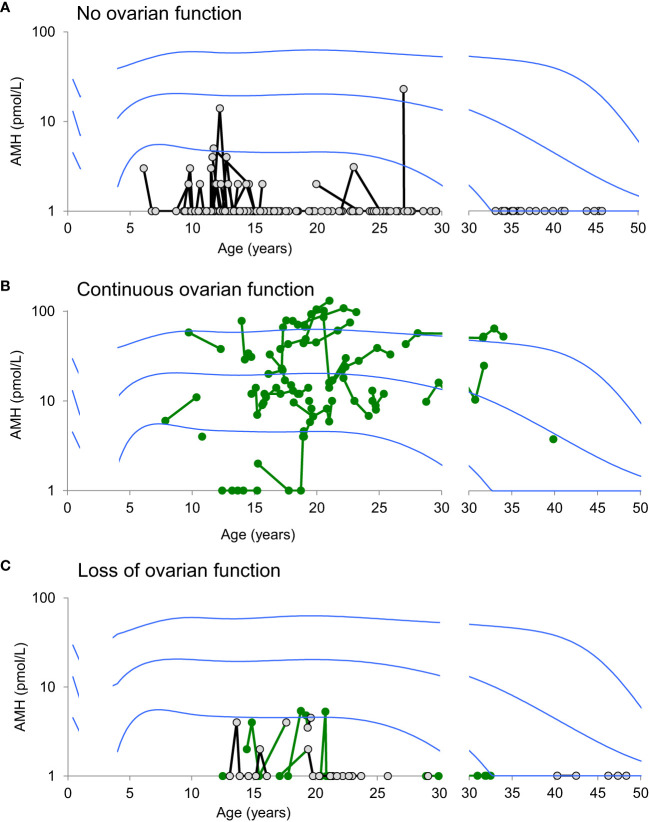
Serum AMH levels (pmol/L) in girls, adolescents, and women with TS according to age, reference range and their ovarian status: never ovarian function **(A)**, ongoing ovarian function **(B)**, and loss of ovarian function **(C)**. Green: patients with ongoing ovarian function; gray/black: patients with no ovarian function. Note the logarithmic Y-scale. Blue lines indicate the reference range (median, 2. and 97.5th percentiles). Figure from (48).

AMH has been associated with ovarian status in adolescent and adult patients with TS; i.e. low or undetectable AMH in patients with POI vs. AMH in the reference range in the majority of patients with ongoing ovarian function (64, 96–99). These cross-sectional data have been confirmed in a longitudinal follow-up study (48) (**Figure 12**). The longitudinal data from TS patients developing POI were sparse and we can therefore not firmly conclude on specific AMH values as predictors of absent pubertal onset or imminent POI. However, AMH was < 5 pmol/L (equals -2 SD) in all patients prior to clinical manifestation of POI. A cross sectional ROC analysis including data from all adolescent and adult patients revealed that AMH < 3 pmol/L seems to be a sensitive and specific marker of POI (both 95%) (48). These findings suggest an increased risk of imminent POI in TS patients with AMH < -2SD. For the clinician, the apparent predictive value of low AMH is useful when counselling adolescent TS patients with ongoing ovarian function about their risk of POI.

**Figure 13:**
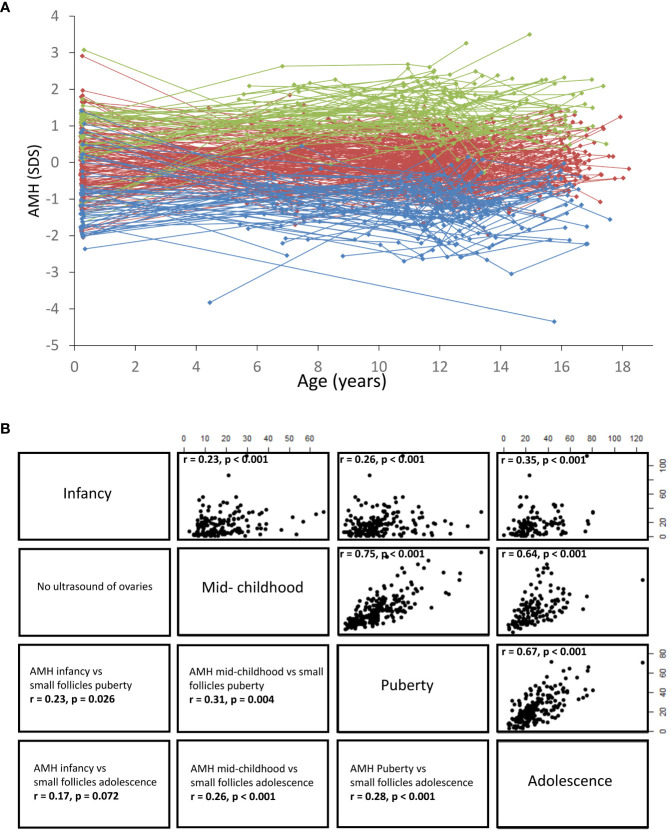
**(A)** Serum AMH concentrations shown as standard deviation scores according to age. Dots indicate individual values and longitudinal courses are connected by lines. All girls were divided into AMH quintiles (5 groups), based on the individual mean SD scores. Blue: 1st quintile, red: 2nd,3rd,4th quintile, green: 5th quintile. **(B)** Right side: Correlations (Spearman´s Rho, r value) between serum AMH concentrations (pmol/L) in infancy, mid-childhood, puberty and adolescence, all p < 0.001. Left side: Correlations (Spearman´s Rho, r value) between serum AMH concentrations and the number of small follicles (<4mm) assessed by transabdominal ultrasound. Figure based on data from (25).

Taking into account that healthy girls maintain their relative AMH levels from infancy to adolescence (68) (**Figure 13**), it seems likely that undetectable AMH or AMH < -2SD is indicative of reduced ovarian activity in prepubertal TS patients. This was supported by our limited longitudinal data on young TS patients where all prepubertal girls with AMH < 4 pmol/L suffered from absent spontaneous pubertal onset (48). These findings are in line with a large European study where girls with TS having measurable AMH had a 19-fold increased chance of entering puberty spontaneously compared with patients with undetectable AMH (96). AMH is also undetectable or low in adult patients suffering from idiopathic premature ovarian insufficiency (100). FSH, LH, inhibin B, and estradiol may be unaffected until time of clinical manifestations of POI where the number of remaining follicles is severely reduced (37, 62, 63, 101). Our findings of multiple undetectable inhibin B measurements as a predictor of absent pubertal onset in young TS patients (47, 102) as well as decreasing inhibin B prior to POI in adolescent and adult patients (48). indicate that also inhibin B may be a valuable predictor of POI. However, single measurements of low or undetectable inhibin B should be interpreted with caution as this is a normal finding in healthy girls and adolescents (103).

Interestingly, adult Turner´s patients with ovarian function maintained their AMH levels during follow up, suggesting that they did not exhibit an accelerated depletion of their ovarian reserve compared to healthy controls (48). This is in line with UK biobank study where women who were not diagnosed with 45,X/46,XX had a similar number of children and did not enter menopause earlier than women with 46,XX (104). Of course there is a risk that the women in this study have a less severe phenotype compared with patients diagnosed with 45,X/46,XX. However, it suggests that patients with 45,X/46,XX have a chance for ongoing ovarian function and unaffected fertility comparable with healthy women. It also underlines the importance of continuous follow-up of such patients.

In conclusion, small studies of patients with TS suggest that AMH < -2SD is predictive of absent puberty and imminent POI, however larger studies are needed to qualify these findings further.

## Ovarian cryopreservation in patients with TS

Hopefully, added understanding of the reproductive phenotype of patients with Turner’s syndrome will lead to an improved evidence-based and individualized fertility counselling. Based on successful experience with ovarian cryopreservation and later auto transplantation in other patients at risk of POI (e.g. girls with cancer prior to gonadotoxic therapy, girls with thalassemia prior to bone marrow transplantation) (105–112), this procedure is now a treatment modality in clinical studies to young patients with TS in several centers. In Sweden, girls with TS have been offered cryopreservation since early 2000´s (50) and in the Netherlands, inclusion of girls with TS in a cryopreservation study has recently been finalized (113). In these studies, many patients had no follicles in the retrieved ovary. Although the karyotype, FSH, AMH, and inhibin B were all associated with the presence of follicles, the sensitivity and specificity of these markers were limited (50).

In this context, it is essential to evaluate ovarian activity. Surgery for ovarian cryopreservation should be avoided in patients without any ovarian follicles. Furthermore, surgery is not indicated in patients with ongoing ovarian function in adult life as they are likely to have a normal prognosis for pregnancy. Knowledge of markers and predictors of ovarian function in girls with TS is essential when counseling patients and their families in these matters. Importantly, studies have shown that life-birth rate after auto transplantation of frozen-thawed ovarian tissue is negatively correlated with increasing age and low AFC, which could indicate that low AMH at the time of cryopreservation plays a prognostic role (114, 115). To date, there are no reports on achieved pregnancies (or live births) in patients with TS after auto transplantation of ovarian tissue.

In prepubertal girls, harvesting of ovarian tissue usually includes laparoscopic retrieval of one of the ovaries inducing a small risk of bleeding and infection. If pregnancy cannot be achieved after auto transplantation, cryopreservation may induce false hope and later psychosocial harm (116). Apart from these ethical issues, the removal of one ovary may potentially cause even earlier loss of valuable ovarian function. Importantly, hidden nests of viable 46,XX oocytes with the potential of future fertilization may get lost.

**Figure 14:**
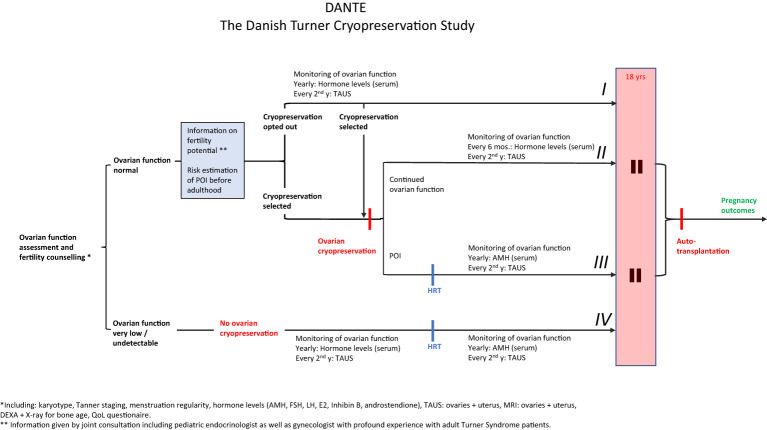
Study design of The Danish Turner Cryopreservation Study (DANTE). All Danish Turner Syndrome patients 2-18 years will be invited to participate. From an initial evaluation of ovarian function (Karyotype, Tanner staging, menstrual cycle regularity, circulating levels of reproductive hormones, transabdominal ultrasound and MRI of the ovaries) we will evaluate if ovarian function is normal or very low/absent. The patient will only be offered ovarian cryopreservation if parameters of ovarian function are within age-matched reference ranges. Based on thorough information concerning fertility potential including estimation of risk of POI before adulthood, the patient will decide for cryopreservation. The patient can opt out cryopreservation and monitor ovarian function closely (I) to select cryopreservation at a later stage if ovarian function declines (II and III). If initial evaluation of ovarian function reveals very low or no ovarian activity, the patients will not be offered cryopreservation (IV). POI (premature ovarian insufficiency): Prepubertal girls: AMH < -2SD and FSH > 2SD, transabdominal ultrasound: streak gonads or antral follicle count (AFC) < 10th percentile (57, 64). Post/ peripubertal: No spontaneous puberty, pubertal progression stopped or markedly delayed (117), or primary/secondary amenorrhea. Furthermore: AMH, E2 and inhibin B < -2SD, FSH > 2SD, streak ovaries and AFC < 10th percentile (57, 64).

Taking these considerations into account, we have designed a national protocol offering selected girls and adolescents with TS ovarian cryopreservation; The Danish Turner Cryopreservation (DANTE) Study (start of inclusion planned in 2023). Ideally, only patients with sufficient numbers of primordial follicles who in the future will experience POI before time of desired pregnancy will benefit from this intervention. In The DANTE Study, all Turner patients (2-18 years) are invited to participate, see Flow-diagram (**Figure 14**). The patient is initially screened for ovarian activity including Tanner staging by physical examination, assessment of circulating concentrations of reproductive hormones (e.g. AMH, FSH, LH, Inhibin B, estradiol), and transabdominal ultrasound of the ovaries to assess the number of antral follicles. If ovarian activity is very low (e.g. AMH < 2SD) or undetectable, the patient is not offered cryopreservation. Prepubertal girls will be followed longitudinally until POI can be confirmed at time of expected puberty.

In case of ovarian activity, the patient and her family receive information at a visit where both the pediatrician and a gynecologist participate. At this meeting we inform of expected fertility potential with and without ovarian cryopreservation based on current knowledge. Based on the initial screening, we will discuss the chances of remaining ovarian function in adult life without intervention, if we expect to find enough follicles by cryopreservation, details about the procedure of future auto transplantation, the success rates in other groups of patients, and we will describe alternative methods of establishing a family (oocyte donation, adoption).

As an alternative strategy for fertility preservation in adolescents and young adults with TS, oocyte vitrification after ovarian stimulation could be considered (118–120). The first live birth after vitrification of oocytes in a woman with TS was recently reported (121).

## Transition clinic

During the past 20 years, we have established joint clinics for adolescent patients in our tertiary center between pediatric endocrinologists and gynecologists as well as adult endocrinologists, as also recommended in the international guideline (122). We have seen nearly 600 patients in these joint transition clinics. Patients with TS are primarily transferred to the gynecological department after adolescence. If they suffer from hypothyroidism or other endocrine conditions, they are also transferred to the department of endocrinology. The pediatrician and the gynecologist/endocrinologist see the patients and their families at a joint consultation in familiar surroundings at the pediatric department one or more times before the age of 18 years. The content of the joint visit is highly individual. Usual topics include e.g. treatment of menstrual irregularities, information on hormone replacement therapy (HRT) including dose and treatment, contraception, sexually transmitted diseases, HPV vaccine, and fertility options. This is also an opportunity to evaluate transabdominal ultrasound of the internal genitalia with special focus on uterine growth by estradiol treatment. The patients are informed about what to expect after the transfer from pediatric to adult follow up. Many adolescents have reservations concerning gynecological examinations, and the transition clinic is an opportunity to stress that this is not a mandatory part of consultations at the gynecological department. We experience that the patients are better prepared and more confident to change to an adult setting, reducing the risk of drop out after referral. However, also the pediatricians and colleagues at the adult departments benefit mutually professionally and scientifically from these joint consultations facilitating sharing of knowledge in rare endocrine disorders, updates on guidelines from other disciplines, novel and emerging treatment options, new evidence, organization of departments, and inspiration to research projects bridging adolescents and young adult patients.

## Summary

Girls with TS are at increased risk of premature ovarian insufficiency. Many of these patients are diagnosed in mid-childhood, but due to central inhibition of the HPG axis, it is difficult to evaluate ovarian activity in girls prior to pubertal onset. Studies of ovarian morphology and reproductive hormones in healthy girls support that AMH is produced by granulosa cells surrounding small ovarian follicles. Even prior to pubertal onset, these follicles are continuously recruited from the pool of primordial follicles independently of gonadotropin-stimulation.

Circulating levels of AMH are predictive of the reproductive lifespan in healthy adult women. Our findings strongly indicate that the inter-individual variation of AMH in girls is indicative of the number of remaining primordial follicles – an important outcome in epidemiological research evaluating factors affecting prenatal establishment of the primordial follicle pool. Despite strong evidence of AMH as a quantitative marker of ovarian follicles, AMH does not predict the specific age at menopause for a given woman, nor is low AMH associated with reduced fecundability in young healthy women.

Marked inter-individual variation but little intra-individual variation of AMH in girls both reflects and predicts the number of small antral follicles. Thus, girls maintain their relative level of ovarian activity from follicles growing independently from FSH stimulation through infancy, childhood, puberty and into adolescence. Limited longitudinal data suggests AMH as a unique predictor of premature ovarian insufficiency in TS patients at risk of accelerated loss of follicles. AMH is therefore a key parameter when counseling patients and their families about future ovarian function. The karyotype of the patient as well as consecutive assessment of circulating levels of inhibin B and FSH may add to the predictive value of ovarian function of a given patient. This information is essential when considering whether the patient could benefit from ovarian cryopreservation.

Thus, the clinical use of AMH has been expanded from a marker of testicular tissue in rare DSD patients to a marker and predictor of ovarian activity used at a daily basis in pediatric endocrinology.

## Author contributions

CH: deciding the topic for the review, drafting the manuscript. MF, GM, TM, LC, CG, MV, AJ, AP: revision of draft. KM: deciding the topic for the review, revision of draft. All authors contributed to the article and approved the submitted version.

## References

[1] Nielsen J Wohlert M. Chromosome abnormalities found among 34,910 newborn children: results from a 13-year incidence study in arhus, Denmark. Hum Genet (1991) 87(1):81–3. doi: 10.1007/BF01213097 2037286

[2] Modi DN Sane S Bhartiya D. Accelerated germ cell apoptosis in sex chromosome aneuploid fetal human gonads. Mol Hum Reprod (2003) 9(4):219–25. doi: 10.1093/molehr/gag031 12651904

[3] Ogata T Matsuo N. Turner syndrome and female sex chromosome aberrations: deduction of the principal factors involved in the development of clinical features. Hum Genet (1995) 95(6):607–29. doi: 10.1007/BF00209476 7789944

[4] Speed RM. Oocyte development in XO foetuses of man and mouse: the possible role of heterologous X-chromosome pairing in germ cell survival. Chromosoma (1986) 94(2):115–24. doi: 10.1007/BF00286989 3757617

[5] Riis ML Nielsen JE Hagen CP de Meyts ER Græm N Jørgensen A. Accelerated loss of oogonia and impaired folliculogenesis in females with turner syndrome start during early fetal development. Hum Reprod (2021) 36(11):2992–3002. doi: 10.1093/humrep/deab210 34568940

[6] Pasquino AM Passeri F Pucarelli I Segni M Municchi G. Spontaneous pubertal development in turner’s syndrome. Italian study group for turner’s syndrome. J Clin Endocrinol Metab (1997) 82(6):1810–3. doi: 10.1210/jcem.82.6.3970 9177387

[7] Baker TG. A quantitative and cytological study of germ cells in human ovaries. Proc R Soc Lond B Biol Sci (1963) 158:417–33. doi: 10.1098/rspb.1963.0055 14070052

[8] Chellakooty M Schmidt IM Haavisto AM Boisen KA Damgaard IN Mau C. Inhibin b, follicle-stimulating hormone, luteinizing hormone, estradiol, and sex hormone-binding globulin levels in 473 healthy infant girls. J Clin Endocrinol Metab (2003) 88(8):3515–20. doi: 10.1210/jc.2002-021468 12915629

[9] Schmidt KLT Kryger-Baggesen N Byskov AG Andersen CY. Anti-müllerian hormone initiates growth of human primordial follicles in vitro. Mol Cell Endocrinol (2005) 234(1–2):87–93. doi: 10.1016/j.mce.2004.12.010 15836957

[10] Frederiksen H Johannsen TH Andersen SE Albrethsen J Landersoe SK Petersen JH. Sex-specific estrogen levels and reference intervals from infancy to late adulthood determined by LC-MS/MS. J Clin Endocrinol Metab (2020) 105(3):754–68. doi: 10.1210/clinem/dgz196 PMC7007877 31720688

[11] Jeppesen JV Anderson RA Kelsey TW Christiansen SL Kristensen SG Jayaprakasan K. Which follicles make the most anti-mullerian hormone in humans? evidence for an abrupt decline in AMH production at the time of follicle selection. Mol Hum Reprod (2013) 19(8):519–27. doi: 10.1093/molehr/gat024 23562944

[12] Jost A. The age factor in the castration of male rabbit fetuses. Proc Soc Exp Biol Med (2023) 66(2):302–3. doi: 10.3181/00379727-66-16071 18921738

[13] Lindhardt Johansen M Hagen CP Johannsen TH Main KM Picard JY Jørgensen A. Anti-müllerian hormone and its clinical use in pediatrics with special emphasis on disorders of sex development. Int J Endocrinol (2013) 2013. doi: 10.1155/2013/198698 PMC3866787 24367377

[14] Rajpert-De Meyts E Jørgensen N Græm N Müller J Cate RL Skakkebæk NE. Expression of anti-müllerian hormone during normal and pathological gonadal development: association with differentiation of sertoli and granulosa cells. J Clin Endocrinol Metab (1999) 84(10):3836–44. doi: 10.1210/jcem.84.10.6047 10523039

[15] Cate RL Mattaliano RJ Hession C Tizard R Farber NM Cheung A. Isolation of the bovine and human genes for müllerian inhibiting substance and expression of the human gene in animal cells. Cell (1986) 45:685–98. doi: 10.1016/0092-8674(86)90783-x 3754790

[16] Josso N di Clemente N. Transduction pathway of anti-müllerian hormone, a sex-specific member of the TGF-β family. Trends Endocrinol Metab (2003) 14(2):91–7. doi: 10.1016/S1043-2760(03)00005-5 12591180

[17] di Clemente N Josso N Gouédard L Belville C. Components of the anti-müllerian hormone signaling pathway in gonads. Mol Cell Endocrinol (2003) 211(1–2):9–14. doi: 10.1016/j.mce.2003.09.005 14656470

[18] Josso N Legeai L Forest MG Chaussain JL Brauner R. An enzyme linked immunoassay for anti-müllerian hormone: a new tool for the evaluation of testicular function in infants and children. J Clin Endocrinol Metab (1990) 70(1):23–7. doi: 10.1210/jcem-70-1-23 1688440

[19] Lee MM Misra M Donahoe PK MacLaughlin DT. MIS/AMH in the assessment of cryptorchidism and intersex conditions. Mol Cell Endocrinol (2003) 211(1–2):91–8. doi: 10.1016/j.mce.2003.09.014 14656481

[20] Lee MM Donahoe PK Silverman BL Hasegawa T Hasegawa Y Gustafson ML. Measurements of serum müllerian inhibiting substance in the evaluation of children with nonpalpable gonads. N Engl J Med (1997) 336(21):1480–6. doi: 10.1056/NEJM199705223362102 9154766

[21] Rey RA Belville C Nihoul-Fékété C Michel-Calemard L Forest MG Lahlou N. Evaluation of gonadal function in 107 intersex patients by means of serum antimüllerian hormone measurement. J Clin Endocrinol Metab (1999) 84(2):627–31. doi: 10.1210/jcem.84.2.5507 10022428

[22] Griesinger G Dafopoulos K Buendgen N Cascorbi I Georgoulias P Zavos A. Elimination half-life of anti-müllerian hormone. J Clin Endocrinol Metab (2012) 97(6):2160–3. doi: 10.1210/jc.2012-1070 22442264

[23] Eilsø Nielsen M Rasmussen IA Fukuda M Westergaard LG Yding Andersen C. Concentrations of anti-müllerian hormone in fluid from small human antral follicles show a negative correlation with CYP19 mRNA expression in the corresponding granulosa cells. Mol Hum Reprod (2010) 16(9):637–43. doi: 10.1093/molehr/gaq001 20064870

[24] Durlinger ALL Gruijters MJG Kramer P Karels B Ingraham HA Nachtigal MW. Anti-müllerian hormone inhibits initiation of primordial follicle growth in the mouse ovary. Endocrinology (2002) 143(3):1076–84. doi: 10.1210/endo.143.3.8691 11861535

[25] Durlinger ALL Gruijters MJG Kramer P Karels B Kumar TR Matzuk MM. Anti-müllerian hormone attenuates the effects of FSH on follicle development in the mouse ovary. Endocrinology (2001) 142(11):4891–9. doi: 10.1210/endo.142.11.8486 11606457

[26] Xu J Bishop CV Lawson MS Park BS Xu F. Anti-müllerian hormone promotes pre-antral follicle growth, but inhibits antral follicle maturation and dominant follicle selection in primates. Hum Reprod (2016) 31(7):1522–30. doi: 10.1093/humrep/dew100 PMC4901882 27165618

[27] Alvaro Mercadal B Imbert R Demeestere I Gervy C de Leener A Englert Y. AMH mutations with reduced in vitro bioactivity are related to premature ovarian insufficiency. Hum Reprod (2015) 30(5):1196–202. doi: 10.1093/humrep/dev042 25750103

[28] Cimino I Casoni F Liu X Messina A Parkash J Jamin SP. Novel role for anti-müllerian hormone in the regulation of GnRH neuron excitability and hormone secretion. Nat Commun (2016) 7. doi: 10.1038/ncomms10055 PMC4729924 26753790

[29] Lane AH Lee MM Fuller AF Kehas DJ Donahoe PK MacLaughlin DT. Diagnostic utility of müllerian inhibiting substance determination in patients with primary and recurrent granulosa cell tumors. Gynecol Oncol (1999) 73(1):51–5. doi: 10.1006/gyno.1998.5290 10094880

[30] Lauritsen MP Bentzen JG Pinborg A Loft A Forman JL Thuesen LL. The prevalence of polycystic ovary syndrome in a normal population according to the Rotterdam criteria versus revised criteria including anti-mullerian hormone. Hum Reprod (2014) 29(4):791–801. doi: 10.1093/humrep/det469 24435776

[31] Pigny P Jonard S Robert Y Dewailly D. Serum anti-mullerian hormone as a surrogate for antral follicle count for definition of the polycystic ovary syndrome. J Clin Endocrinol Metab (2006) 91(3):941–5. doi: 10.1210/jc.2005-2076 16368745

[32] Randolph JF Harlow SD Helmuth ME Zheng H McConnell DS. Updated assays for inhibin b and AMH provide evidence for regular episodic secretion of inhibin b but not AMH in the follicular phase of the normal menstrual cycle. Hum Reprod (2014) 29(3):592–600. doi: 10.1093/humrep/det447 24357435 PMC3923509

[33] Fanchin R Taieb J Lozano DHM Ducot B Frydman R Bouyer J. High reproducibility of serum anti-mullerian hormone measurements suggests a multi-staged follicular secretion and strengthens its role in the assessment of ovarian follicular status. Hum Reprod (2005) 20(4):923–7. doi: 10.1093/humrep/deh688 15640257

[34] van Disseldorp J Lambalk CB Kwee J Looman CWN Eijkemans MJC Fauser BC. Comparison of inter- and intra-cycle variability of anti-mullerian hormone and antral follicle counts. Hum Reprod (2010) 25(1):221–7. doi: 10.1093/humrep/dep366 19840990

[35] Bentzen JG Forman JL Pinborg A Lidegaard O Larsen EC Friis-Hansen L. Ovarian reserve parameters: a comparison between users and non-users of hormonal contraception. Reprod BioMed (2012) 25(6):612–9. doi: 10.1016/j.rbmo.2012.09.001 23069740

[36] Streuli I Fraisse T Pillet C Ibecheole V Bischof P de Ziegler D. Serum antimüllerian hormone levels remain stable throughout the menstrual cycle and after oral or vaginal administration of synthetic sex steroids. Fertil Steril (2008) 90(2):395–400. doi: 10.1016/j.fertnstert.2007.06.023 17919608

[37] de Vet A Laven JSE de Jong FH Themmen APN Fauser BCJM. Antimüllerian hormone serum levels: a putative marker for ovarian aging. Fertil Steril (2002) 77(2):357–62. doi: 10.1016/S0015-0282(01)02993-4 11821097

[38] Gougeon A. Ovarian follicular growth in humans: ovarian ageing and population of growing follicles. Maturitas (1998) 30(2):137–42. doi: 10.1016/S0378-5122(98)00069-3 9871908

[39] Gougeon A Ecochard R Thalabard JC. Age-related changes of the population of human ovarian follicles: increase in the disappearance rate of non-growing and early-growing follicles in aging women. Biol Reprod (1994) 50(3):653–63. doi: 10.1095/biolreprod50.3.653 8167237

[40] Hansen KR Hodnett GM Knowlton N Craig LB. Correlation of ovarian reserve tests with histologically determined primordial follicle number. Fertil Steril (2011) 95(1):170–5. doi: 10.1016/j.fertnstert.2010.04.006 20522327

[41] Tehrani FR Solaymani-Dodaran M Tohidi M Gohari MR Azizi F. Modeling age at menopause using serum concentration of anti-mullerian hormone. J Clin Endocrinol Metab (2013) 98(2):729–35. doi: 10.1210/jc.2012-3176 23316087

[42] Tehrani FR Shakeri N Solaymani-Dodaran M Azizi F. Predicting age at menopause from serum antimüllerian hormone concentration. Menopause (2011) 18(7):766–70. doi: 10.1097/gme.0b013e318205e2ac 21451424

[43] Freeman EW Sammel MD Lin H Gracia CR. Anti-mullerian hormone as a predictor of time to menopause in late reproductive age women. J Clin Endocrinol Metab (2012) 97(5):1673–80. doi: 10.1210/jc.2011-3032 PMC3339896 22378815

[44] Dólleman M Verschuren WMM Eijkemans MJC Broekmans FJM van der Schouw YT. Added value of anti-müllerian hormone in prediction of menopause: results from a large prospective cohort study. Hum Reprod (2015) 30(8):1974–81. doi: 10.1093/humrep/dev145 26082477

[45] Broer SL Eijkemans MJC Scheffer GJ van Rooij IAJ de Vet A Themmen APN. Anti-mullerian hormone predicts menopause: a long-term follow-up study in normoovulatory women. J Clin Endocrinol Metab (2011) 96(8):2532–9. doi: 10.1210/jc.2010-2776 21613357

[46] Nelson SM Davis SR Kalantaridou S Lumsden MA Panay N Anderson RA. Anti-müllerian hormone for the diagnosis and prediction of menopause: a systematic review. Hum Reprod (2023) 29:327–46. doi: 10.1093/humupd/dmac045 PMC10152172 36651193

[47] Hagen CP Main KM Kjaergaard S Juul AFSH. LH, inhibin b and estradiol levels in turner syndrome depend on age and karyotype: longitudinal study of 70 turner girls with or without spontaneous puberty. Hum Reprod (2010) 25(12):3134–41. doi: 10.1093/humrep/deq291 20956269

[48] Lunding SA Aksglaede L Anderson RA Main KM Juul A Hagen CP. AMH as predictor of premature ovarian insufficiency: a longitudinal study of 120 turner syndrome patients. J Clin Endocrinol Metab (2015) 100(7):E1030–8. doi: 10.1210/jc.2015-1621 25978111

[49] Reynaud K Cortvrindt R Verlinde F de Schepper J Bourgain C Smitz J. Number of ovarian follicles in human fetuses with the 45,X karyotype. Fertil Steril (2004) 81(4):1112–9. doi: 10.1016/j.fertnstert.2003.12.011 15066472

[50] Birgit B Julius H Carsten R Maryam S Gabriel F Victoria K. Fertility preservation in girls with turner syndrome: prognostic signs of the presence of ovarian follicles. J Clin Endocrinol Metab (2009) 94(1):74–80. doi: 10.1210/jc.2008-0708 18957497

[51] Ogata T Muroya K Matsuo N Shinohara O Yorifuji T Nishi Y. Turner syndrome and xp deletions: clinical and molecular studies in 47 patients. J Clin Endocrinol Metab (2001) 86(11):5498–508. doi: 10.1210/jcem.86.11.8058 11701728

[52] Hook EB Warburton D. The distribution of chromosomal genotypes associated with turner’s syndrome: livebirth prevalence rates and evidence for diminished fetal mortality and severity in genotypes associated with structural X abnormalities or mosaicism. Hum Genet (1983) 64(1):24–7. doi: 10.1007/BF00289473 6683706

[53] Peek R Schleedoorn M Smeets D van de Zande G Groenman F Braat D. Ovarian follicles of young patients with turner’s syndrome contain normal oocytes but monosomic 45,X granulosa cells. Hum Reprod (2019) 34(9):1686–96. doi: 10.1093/humrep/dez135 PMC6736193 31398245

[54] Lespinasse J Gicquel C Robert M le Bouc Y. Phenotypic and genotypic variability in monozygotic triplets with turner syndrome. Clin Genet (1998) 54(1):56–9. doi: 10.1111/j.1399-0004.1998.tb03694.x 9727741

[55] Nadesapillai S van der Velden J Smeets D van de Zande G Braat D Fleischer K. Why are some patients with 45,X turner syndrome fertile? a young girl with classical 45,X turner syndrome and a cryptic mosaicism in the ovary. Fertil Steril (2021) 115(5):1280–7. doi: 10.1016/j.fertnstert.2020.11.006 33342535

[56] Mortensen KH Rohde MD Uldbjerg N Gravholt CH. Repeated spontaneous pregnancies in 45,X turner syndrome. Obstetrics gynecology (2010) 115(2 Pt 2):446–9. doi: 10.1097/AOG.0b013e3181cb5b2a 20093875

[57] Hagen CP Mouritsen A Mieritz MG Tinggaard J Wohlfart-Veje C Fallentin E. Circulating AMH reflects ovarian morphology by magnetic resonance imaging and 3D ultrasound in 121 healthy girls. J Clin Endocrinol Metab (2015) 100(3):880–90. doi: 10.1210/jc.2014-3336 25485726

[58] Conte FA Grumbach MM Kaplan SL. A diphasic pattern of gonadotropin secretion in patients with the syndrome of gonadal dysgenesis. J Clin Endocrinol Metab (1975) 40(4):670–4. doi: 10.1210/jcem-40-4-670 1127077

[59] Fechner PY Davenport ML Qualy RL Ross JL Gunther DF Eugster EA. Differences in follicle-stimulating hormone secretion between 45,X monosomy turner syndrome and 45,X/46,XX mosaicism are evident at an early age. J Clin Endocrinol Metab (2006) 91(12):4896–902. doi: 10.1210/jc.2006-1157 16968797

[60] Mouritsen A Aksglaede L Soerensen K Hagen CP Petersen JH Main KM. The pubertal transition in 179 healthy Danish children: associations between pubarche, adrenarche, gonadarche, and body composition. Eur J Endocrinol (2012) 168(2):129–36. doi: 10.1530/EJE-12-0191 23093700

[61] Metcalf MG Skidmore DS Lowry GF Mackenzie JA. Incidence of ovulation in the years after the menarche. J Endocrinol (1983) 97(2):213–9. doi: 10.1677/joe.0.0970213 6854190

[62] Fanchin R Schonäuer LM Righini C Guibourdenche J Frydman R Taieb J. Serum anti-müllerian hormone is more strongly related to ovarian follicular status than serum inhibin b, estradiol, FSH and LH on day 3. Hum Reprod (2003) 18(2):323–7. doi: 10.1093/humrep/deg042 12571168

[63] van Rooij IAJ den Tonkelaar I Broekmans FJM Looman CWN Scheffer GJ de Jong FH. Anti-müllerian hormone is a promising predictor for the occurrence of the menopausal transition. Menopause (2004) 11(6 Pt 1):601–6. doi: 10.1097/01.GME.0000123642.76105.6E 15545787

[64] Hagen CP Aksglaede L Sørensen K Main KM Boas M Cleemann L. Serum levels of anti-müllerian hormone as a marker of ovarian function in 926 healthy females from birth to adulthood and in 172 turner syndrome patients. J Clin Endocrinol Metab (2010) 95(11):5003–10. doi: 10.1210/jc.2010-0930 20719830

[65] Ljubicic ML Busch AS Upners E Fischer MB Petersen JH Raket LL. Biphasic pattern of circulating reproductive hormones in female infants - the longitudinal COPENHAGEN minipuberty study. Horm Res Paediatr (2021) 94(Suppl. 1):24–5. doi: 10.1210/clinem/dgac363

[66] Kuiri-Hänninen T Kallio S Seuri R Tyrväinen E Liakka A Tapanainen J. Postnatal developmental changes in the pituitary-ovarian axis in preterm and term infant girls. J Clin Endocrinol Metab (2011) 96(11):3432–9. doi: 10.1210/jc.2011-1502 21900380

[67] Ersen A Onal H Yildirim D Adal E. Ovarian and uterine ultrasonography and relation to puberty in healthy girls between 6 and 16 years in the Turkish population: a cross-sectional study. J Pediatr Endocrinol Metab. (2012) 25(5–6):447–51. doi: 10.1515/jpem-2012-0014 22876537

[68] Hagen CP Fischer MB Wohlfahrt-Veje C Assens M Busch AS Pedersen AT. AMH concentrations in infancy and mid-childhood predict ovarian activity in adolescence: a long-term longitudinal study of healthy girls. EClinicalMedicine (2023) 55:101742. doi: 10.1016/j.eclinm.2022.101742 36386030 PMC9661496

[69] Kelsey TW Wright P Nelson SM Anderson RA Wallace WHB. A validated model of serum anti-müllerian hormone from conception to menopause. PloS One (2011) 6(7):E22024. doi: 10.1371/journal.pone.0022024 21789206 PMC3137624

[70] Rustamov O Smith A Roberts SA Yates AP Fitzgerald C Krishnan M. The measurement of anti-müllerian hormone: a critical appraisal. J Clin Endocrinol Metab (2014) 99(3):723–32. doi: 10.1210/jc.2013-3476 24423305

[71] Rustamov O Smith A Roberts SA Yates AP Fitzgerald C Krishnan M. Anti-mullerian hormone: poor assay reproducibility in a large cohort of subjects suggests sample instability. Hum Reprod (2012) 27(10):3085–91. doi: 10.1093/humrep/des260 22777530

[72] Mamsen LS Petersen TS Jeppesen JV Mollgård K Grondahl ML Larsen A. Proteolytic processing of anti-müllerian hormone differs between human fetal testes and adult ovaries. Mol Hum Reprod (2015) 21(7):571–82. doi: 10.1093/molehr/gav024 25920489

[73] Pankhurst MW Chong YH McLennan IS. Enzyme-linked immunosorbent assay measurements of antimüllerian hormone (AMH) in human blood are a composite of the uncleaved and bioactive cleaved forms of AMH. Fertil Steril (2014) 101(3):846–50. doi: 10.1016/j.fertnstert.2013.12.009 24424371

[74] Hagen CP Aksglaede L Sørensen K Mouritsen A Andersson AM Petersen JH. Individual serum levels of anti-müllerian hormone in healthy girls persist through childhood and adolescence: a longitudinal cohort study. Hum Reprod (2012) 27(3):861–6. doi: 10.1093/humrep/der435 22215627

[75] Lashen H Dunger DB Ness A Ong KK. Peripubertal changes in circulating antimüllerian hormone levels in girls. Fertil Steril (2013) 99(7):2071–5. doi: 10.1016/j.fertnstert.2013.01.139 PMC3906604 23419927

[76] Jeffery A Streeter AJ Hosking J Wilkin TJ Nelson SM. Anti-müllerian hormone in children: a ten-year prospective longitudinal study (EarlyBird 39). J Pediatr Endocrinol Metab (2015) 28(9–10):1153–62. doi: 10.1515/jpem-2014-0517 26030784

[77] Grynberg M Pierre A Rey R Leclerc A Arouche N Hesters L. Differential regulation of ovarian anti-müllerian hormone (AMH) by estradiol through α- and β-estrogen receptors. J Clin Endocrinol Metab (2012) 97(9):E1649–57. doi: 10.1210/jc.2011-3133 22689696

[78] Pellatt L Rice S Dilaver N Heshri A Galea R Brincat M. Anti-müllerian hormone reduces follicle sensitivity to follicle-stimulating hormone in human granulosa cells. Fertil Steril (2011) 96(5):1246–51. doi: 10.1016/j.fertnstert.2011.08.015 21917251

[79] Pellatt L Hanna L Brincat M Galea R Brain H Whitehead S. Granulosa cell production of anti-müllerian hormone is increased in polycystic ovaries. J Clin Endocrinol Metab (2007) 92(1):240–5. doi: 10.1210/jc.2006-1582 17062765

[80] Somunkiran A Yavuz T Yucel O Ozdemir I. Anti-müllerian hormone levels during hormonal contraception in women with polycystic ovary syndrome. Eur J Obstet Gynecol Reprod Biol (2007) 134(2):196–201. doi: 10.1016/j.ejogrb.2007.01.012 17335955

[81] Kallio S Puurunen J Ruokonen A Vaskivuo T Piltonen T Tapanainen JS. Antimüllerian hormone levels decrease in women using combined contraception independently of administration route. Fertil Steril (2013) 99(5):1305–10. doi: 10.1016/j.fertnstert.2012.11.034 23260855

[82] Dolleman M Faddy MJ Disseldorp Jv Schouw YTvd Messow CM Leader B. The relationship between anti-müllerian hormone in women receiving fertility assessments and age at menopause in subfertile women: evidence from large population studies. J Clin Endocrinol Metab (2013) 98(5):1946–53. doi: 10.1210/jc.2012-4228 23509105

[83] Hagen CP Sorensen K Anderson RA Juul A. Serum levels of antimüllerian hormone in early maturing girls before, during, and after suppression with GnRH agonist. Fertil Steril (2012) 98(5):1326–30. doi: 10.1016/j.fertnstert.2012.07.1118 22901847

[84] Jensen AMB Brocks V Holm K Laursen EM Muller J. Central precocious puberty in girls: internal genitalia before, during, and after treatment with long-acting gonadotropin-releasing hormone analogues. J Pediatr (1998) 132(1):105–8. doi: 10.1016/S0022-3476(98)70493-7 9470009

[85] Broer SL Mol BWJ Hendriks D Broekmans FJM. The role of antimullerian hormone in prediction of outcome after IVF: comparison with the antral follicle count. Fertil Steril (2009) 91(3):705–14. doi: 10.1016/j.fertnstert.2007.12.013 18321493

[86] Broer SL van Disseldorp J Broeze KA Dolleman M Opmeer BC Bossuyt P. Added value of ovarian reserve testing on patient characteristics in the prediction of ovarian response and ongoing pregnancy: an individual patient data approach. Hum Reprod Update (2013) 19(1):26–36. doi: 10.1093/humupd/dms041 23188168

[87] Tal R Tal O Seifer BJ Seifer DB. Antimüllerian hormone as predictor of implantation and clinical pregnancy after assisted conception: a systematic review and meta-analysis. Fertil Steril (2015) 103(1):119–130.e3. doi: 10.1016/j.fertnstert.2014.09.041 25450298

[88] Khader A Lloyd SM McConnachie A Fleming R Grisendi V la Marca A. External validation of anti-müllerian hormone based prediction of live birth in assisted conception. J Ovarian Res (2013) 6(1). doi: 10.1186/1757-2215-6-3 PMC3546900 23294733

[89] la Marca A Nelson SM Sighinolfi G Manno M Baraldi E Roli L. Anti-müllerian hormone-based prediction model for a live birth in assisted reproduction. Reprod BioMed Online (2011) 22(4):341–9. doi: 10.1016/j.rbmo.2010.11.005 21317041

[90] Steiner AZ Herring AH Kesner JS Meadows JW Stanczyk FZ Hoberman S. Antimüllerian hormone as a predictor of natural fecundability in women aged 30-42 years. Obstetrics gynecology (2011) 117(4):798–804. doi: 10.1097/AOG.0b013e3182116bc8 21422850 PMC3825553

[91] Casadei L Manicuti C Puca F Madrigale A Emidi E Piccione E. Can anti-müllerian hormone be predictive of spontaneous onset of pregnancy in women with unexplained infertility? J Obstet Gynaecol (2013) 33(8):857–61. doi: 10.3109/01443615.2013.831050 24219729

[92] Hagen CP Vestergaard S Juul A Skakkebæk NE Andersson AM Main KM. Low concentration of circulating antimüllerian hormone is not predictive of reduced fecundability in young healthy women: a prospective cohort study. Fertil Steril (2012) 98(6):1602–8. doi: 10.1016/j.fertnstert.2012.08.008 22959460

[93] Zarek SM Mitchell EM Sjaarda LA Mumford SL Silver RM Stanford JB. Is anti-müllerian hormone associated with fecundability? findings from the EAGeR trial. J Clin Endocrinol Metab (2015) 100(11):4215–21. doi: 10.1210/jc.2015-2474 PMC4702454 26406293

[94] Streuli I de Mouzon J Paccolat C Chapron C Petignat P Irion OP. AMH concentration is not related to effective time to pregnancy in women who conceive naturally. Reprod BioMed Online (2014) 28(2):216–24. doi: 10.1016/j.rbmo.2013.10.007 24365018

[95] Qiu W Luo K Lu Y Zhao J Wang Y Yang H. Anti-müllerian hormone has limited ability to predict fecundability in Chinese women: a preconception cohort study. Reprod BioMed Online (2022) 44(6):1055–63. doi: 10.1016/j.rbmo.2022.02.014 35461761

[96] Visser JA Hokken-Koelega ACS Zandwijken GRJ Limacher A Ranke MB Flück CE. Anti-müllerian hormone levels in girls and adolescents with turner syndrome are related to karyotype, pubertal development and growth hormone treatment. Hum Reprod (2013) 28(7):1899–907. doi: 10.1093/humrep/det089 23539612

[97] Purushothaman R Lazareva O Oktay K Ten S. Markers of ovarian reserve in young girls with turner’s syndrome. Fertil Steril (2010) 94(4):1557–9. doi: 10.1016/j.fertnstert.2009.12.026 20097335

[98] Hamza RT Mira MF Hamed AI Ezzat T Sallam MT. Anti-müllerian hormone levels in patients with turner syndrome: relation to karyotype, spontaneous puberty, and replacement therapy. Am J Med Genet A (2018) 176(9):1929–34. doi: 10.1002/ajmg.a.40473 30088853

[99] Bernard V Donadille B Zenaty D Courtillot C Salenave S Brac de la Perrière A. Spontaneous fertility and pregnancy outcomes amongst 480 women with turner syndrome. Hum Reprod (2016) 31(4):782–8. doi: 10.1093/humrep/dew012 26874361

[100] Knauff EAH Eijkemans MJC Lambalk CB ten Kate-Booij MJ Hoek A Beerendonk CCM. Anti-mullerian hormone, inhibin b, and antral follicle count in young women with ovarian failure. J Clin Endocrinol Metab (2009) 94(3):786–92. doi: 10.1210/jc.2008-1818 19066296

[101] Burger HG Dudley EC Hopper JL Groome N Guthrie JR Green A. Prospectively measured levels of serum follicle-stimulating hormone, estradiol, and the dimeric inhibins during the menopausal transition in a population-based cohort of women. J Clin Endocrinol Metab (1999) 84(11):4025–30. doi: 10.1210/jcem.84.11.6158 10566644

[102] Gravholt CH Naeraa RW Andersson AM Christiansen JS Skakkebæk NE. Inhibin a and b in adolescents and young adults with turner’s syndrome and no sign of spontaneous puberty. Hum Reprod (2002) 17(8):2049–53. doi: 10.1093/humrep/17.8.2049 12151435

[103] Sehested A Juul A Andersson AM Petersen JH Jensen TK Müller J. Serum inhibin a and inhibin b in healthy prepubertal, pubertal, and adolescent girls and adult women: relation to age, stage of puberty, menstrual cycle, follicle-stimulating hormone, luteinizing hormone, and estradiol levels. J Clin Endocrinol Metab (2000) 85(4):1634–40. doi: 10.1210/jcem.85.4.6512 10770209

[104] Tuke MA Ruth KS Wood AR Beaumont RN Tyrrell J Jones SE. Mosaic turner syndrome shows reduced penetrance in an adult population study. Genet Med (2019) 21(4):877–86. doi: 10.1038/s41436-018-0271-6 PMC6752315 30181606

[105] Pfeifer S Goldberg J Lobo R Pisarska M Thomas M Widra E. Ovarian tissue cryopreservation: a committee opinion. Fertil Steril (2014) 101(5):1237–43. doi: 10.1016/j.fertnstert.2014.02.052 24684955

[106] Loren AW Mangu PB Beck LN Brennan L Magdalinski AJ Partridge AH. Fertility preservation for patients with cancer: American society of clinical oncology clinical practice guideline update. J Clin Oncol (2013) 31(19):2500–10. doi: 10.1200/JCO.2013.49.2678 PMC5321083 23715580

[107] Coccia PF Pappo AS Beaupin L Borges VF Borinstein SC Chugh R. Adolescent and young adult oncology, version 2. 2018, NCCN clinical practice guidelines in oncology. J Natl Compr Canc Netw (2018) 16(1):66–97. doi: 10.6004/jnccn.2018.0001 29295883

[108] von Wolff M Montag M Dittrich R Denschlag D Nawroth F Lawrenz B. Fertility preservation in women–a practical guide to preservation techniques and therapeutic strategies in breast cancer, hodgkin’s lymphoma and borderline ovarian tumours by the fertility preservation network FertiPROTEKT. Arch Gynecol Obstet (2011) 284(2):427–35. doi: 10.1007/s00404-011-1874-1 PMC3133651 21431846

[109] Fallat ME Hutter J. Preservation of fertility in pediatric and adolescent patients with cancer. Pediatrics (2008) 121(5):1461–9. doi: 10.1542/peds.2008-0593 18450888

[110] Lambertini M del Mastro L Pescio MC Andersen CY Azim HA Peccatori FA. Cancer and fertility preservation: international recommendations from an expert meeting. BMC Med (2016) 14(1). doi: 10.1186/s12916-015-0545-7 PMC4700580 26728489

[111] Jakes AD Marec-Berard P Phillips RS Stark DP. Critical review of clinical practice guidelines for fertility preservation in teenagers and young adults with cancer. J Adolesc Young Adult Oncol (2014) 3(4):144–52. doi: 10.1089/jayao.2014.0032 PMC4270154 25538859

[112] Mamsen LS Kristensen SG Pors SE Bøtkjær JA Ernst E Macklon KT. Consequences of β-thalassemia or sickle cell disease for ovarian follicle number and morphology in girls who had ovarian tissue cryopreserved. Front Endocrinol (Lausanne) (2021) 11. doi: 10.3389/fendo.2020.593718 PMC7844814 33519708

[113] Schleedoorn M van der Velden J Braat D Beerendonk I van Golde R Peek R. TurnerFertility trial: PROTOCOL for an observational cohort study to describe the efficacy of ovarian tissue cryopreservation for fertility preservation in females with turner syndrome. BMJ Open (2019) 9(12). doi: 10.1136/bmjopen-2019-030855 PMC6924773 31831533

[114] Lotz L Bender-Liebenthron J Dittrich R Häberle L Beckmann MW Germeyer A. Determinants of transplantation success with cryopreserved ovarian tissue: data from 196 women of the FertiPROTEKT network. Hum Reprod (2022) 37(12):2787–96. doi: 10.1093/humrep/deac225 36272106

[115] Colmorn LB Pedersen AT Larsen EC Hansen AS Rosendahl M Andersen CY. Reproductive and endocrine outcomes in a cohort of Danish women following auto-transplantation of Frozen/Thawed ovarian tissue from a single center. Cancers (Basel) (2022) 14(23):5873. doi: 10.3390/cancers14235873 36497354 PMC9740843

[116] van der Coelen S van der Velden J Nadesapillai S Peek R Braat D Schleedoorn M. The decision-making process regarding ovarian tissue cryopreservation in girls with turner syndrome by patients, parents, and healthcare providers: a mixed-methods study. Horm Res Paediatr (2022) 95(4):374–83. doi: 10.1159/000525374 PMC9677842 35671713

[117] Johansen ML Hagen CP Mieritz MG Wolthers OD Heuck C Petersen JH. Pubertal progression and reproductive hormones in healthy girls with transient thelarche. J Clin Endocrinol Metab (2017) 102(3):1001–8. doi: 10.1210/jc.2016-2871 28009526

[118] Huang JYJ Tulandi T Holzer H Lau NM MacDonald S Tan SL. Cryopreservation of ovarian tissue and in vitro matured oocytes in a female with mosaic turner syndrome: case report. Hum Reprod (2008) 23(2):336–9. doi: 10.1093/humrep/dem307 18056118

[119] Kavoussi SK Fisseha S Smith YR Smith GD Christman GM Gago LA. Oocyte cryopreservation in a woman with mosaic turner syndrome: a case report. J Reprod Med (2008) 53(3):223–6. 18441731

[120] Oktay K Rodriguez-Wallberg KA Sahin G. Fertility preservation by ovarian stimulation and oocyte cryopreservation in a 14-year-old adolescent with turner syndrome mosaicism and impending premature ovarian failure. Fertil Steril (2010) 94(2):753.e15–753.e19. doi: 10.1016/j.fertnstert.2010.01.044 20188362

[121] Strypstein L van Moer E Nekkebroeck J Segers I Tournaye H Demeestere I. First live birth after fertility preservation using vitrification of oocytes in a woman with mosaic turner syndrome. J Assist Reprod Genet (2022) 39(2):543–9. doi: 10.1007/s10815-022-02420-4 PMC8956750 35122176

[122] Gravholt CH Andersen NH Conway GS Dekkers OM Geffner ME Klein KO. Clinical practice guidelines for the care of girls and women with turner syndrome: proceedings from the 2016 Cincinnati international turner syndrome meeting. Eur J Endocrinol (2017) 177(3):G1–70. doi: 10.1530/EJE-17-0430 28705803

